# *Cis* Association of Galectin-9 with Tim-3 Differentially Regulates IL-12/IL-23 Expressions in Monocytes via TLR Signaling

**DOI:** 10.1371/journal.pone.0072488

**Published:** 2013-08-14

**Authors:** Cheng J. Ma, Guang Y. Li, Yong Q. Cheng, Jia M. Wang, Ruo S. Ying, Lei Shi, Xiao Y. Wu, Toshiro Niki, Mitsumi Hirashima, Chuan F. Li, Jonathan P. Moorman, Zhi Q. Yao

**Affiliations:** 1 Hepatitis (HCV/HIV) Program, Department of Veterans Affairs, James H. Quillen Veterans Affairs Medical Center, Johnson City, Tennessee, United States of America; 2 Department of Internal Medicine, Division of Infectious Diseases, Quillen College of Medicine, East Tennessee State University, Johnson City, Tennessee, United States of America; 3 International Center for Diagnosis and Treatment of Liver Diseases, 302 Hospital, Beijing, China; 4 Department of Biochemistry and Molecular Biology, Soochow University School of Medicine, Suzhou, China; 5 Department of Hepatology, Guangzhou Number 8 People’s Hospital, Guangzhou, China; 6 Department of Infectious Diseases, Xian Jiaotong University College of Medicine, Xi'an, China; 7 Department of Immunology and Immunopathology, Faculty of Medicine, Kagawa University, Kagawa, Japan; 8 GalPharma, Kagawa, Japan; 9 Department of Surgery, Quillen College of Medicine, East Tennessee State University, Johnson City, Tennessee, United States of America; National Institute of Infectious Diseases, Japan

## Abstract

Human monocytes/macrophages (M/M_Ф_) of the innate immunity sense and respond to microbial products via specific receptor coupling with stimulatory (such as TLR) and inhibitory (such as Tim-3) receptors. Current models imply that Tim-3 expression on M/M_Ø_ can deliver negative signaling to TLR-mediated IL-12 expression through *trans* association with its ligand Galectin-9 (Gal-9) presented by other cells. However, Gal-9 is also expressed within M/M_Ø_, and the effect of intracellular Gal-9 on Tim-3 activities and inflammatory responses in the same M/M_Ø_ remains unknown. In this study, our data suggest that Tim-3 and IL-12/IL-23 gene transcriptions are regulated by enhanced or silenced Gal-9 expression within monocytes through synergizing with TLR signaling. Additionally, TLR activation facilitates Gal-9/Tim-3 *cis* association within the same M/M_Ø_ to differentially regulate IL-12/IL-23 expressions through STAT-3 phosphorylation. These results reveal a ligand (Gal-9) compartment-dependent regulatory effect on receptor (Tim-3) activities and inflammatory responses via TLR pathways—a novel mechanism underlying cellular responses to external or internal cues.

## Introduction

Human monocytes/macrophages (M/M_Ф_) of the innate immune system, the first line of host defense against pathogen infection, sense and respond to microbial signature molecules, known as pathogen associated molecular patterns (PAMPs), via pathogen recognition receptors (PRRs), including toll-like receptors (TLRs), RIG-I-like receptors (RLRs), NOD-like receptors (NLRs), and cytosolic DNA receptors (sensors for DNA) [1]. Recognition of PAMPs by PRRs rapidly triggers an array of anti-microbial responses through the induction of various inflammatory cytokines, chemokines, and type 1 interferons. For example, upon binding PAMPs, TLR signaling involves cross-linking of adapter proteins (such as MyD88, TRIF) and activation of protein kinase pathways to initiate NF_К_B-dependent/-independent (IRF3) gene transcription and cell activation [1]. Activated M/M_Ф_ produce inflammatory cytokines (such as IL-12 and IL-23), which are important for the next phase of host defense against infections [2–6].

IL-12, a heterodimeric cytokine composed of p40 and p35 subunits, is a key cytokine linking innate to adaptive immunity, critical for clearance of acute infection [2,3]. How IL-12 endows T cells with such potential is unclear, but the discovery of IL-23, a heterodimer of IL-12p40 and a unique IL-23p19 subunit, offers new insights into their roles in the pathogenesis of chronic infection and autoimmune disorders [4–6]. Although IL-12 and IL-23 belong to the same family and share a common chain p40, their expressions in M/M_Ф_ are differentially regulated by PAMPs and their functions in polarization of T cells are distinct and often antithetical [2–6]. IL-12 induces IL-2 and IFN-γ expressions that promote polarization toward a T_H_1 rather than T_H_2 response [2,3]. IL-23 induces differentiation of IL-17-producing CD4^+^ helper T cells (T_H_17) and CD4^+^CD25^+^Foxp3^+^ regulatory T cells (Tregs) that favor the development of chronic infection and autoimmune diseases under pathogenic conditions [4–6].

In addition to stimulatory signaling, the host immune system has developed multiple negative regulatory pathways to prevent unnecessary immune responses. Among them, the T cell immunoglobulin mucin domain-3 (Tim-3)/galectin-9 (Gal-9) inhibitory pathway represents such a mechanism to maintain the balance between positive and negative signals in immune responses following antigenic encounter [7,8]. Tim-3 is a type 1 membrane protein with a structurally conserved immunoglobulin variable (IgV) domain and a mucin stalk that anchors to an intracellular tail with SH2 phosphorylation domain [7,8]. Initially identified as preferentially expressed on activated T_H_1 cells, but not T_H_2 cells [9], Tim-3 has since been found to be expressed on and to have more complex functions in other type of cells including M/M_Ф_ [10–16]. While Tim-3 has been shown to play a pivotal role in T cell dysregulation [17–25], its role in innate M/M_Ø_ as well as maturation and function of dendritic cells (DC) is rather controversial [10–16]. On the one hand, Tim-3 negatively regulates macrophage activation, and Tim-3 signaling in cells of the innate immune system critically influences regulation of adaptive immune responses [15,16]. On the other hand, Tim-3 and its ligand Gal-9 induce maturation of DC and promote phagocytosis of apoptotic cells, presenting dying cell-associated antigen to T cells [10–14].

Gal-9 belongs to the tandem-repeat type subfamily of galectins, structurally characterized by the presence of two distinct carbohydrate recognition domains with different sugar binding specificities joined by a linker peptide [26]. Gal-9 exhibits various biological activities such as chemo-attraction, cell aggregation, cell proliferation, cell survival, and immunomodulation of inflammation [26]. Since Gal-9 is widely expressed in various types of cells including M/M_Ф_, an intriguing question is whether extracellular and intracellular Gal-9 functions differently by modulating different signaling pathways. Indeed, we and others have shown that extracellular Gal-9, either in soluble form or presented by other cells, is inhibitory and pro-apoptotic via binding to Tim-3 expressed on M/M_Ф_ in *trans* [15,16,27–30]. Conversely, intracellular Gal-9 rapidly translocates into the nucleus of monocytes upon LPS stimulation, activating IL-1β and IFN-γ gene transcriptions by interaction with nuclear factor-interleukin 6 (NF-IL6) [31]. However, the mechanism(s) involved in regulation of Tim-3 and inflammatory response by intracellular Gal-9 in M/M_Ø_ remains elusive.

In this study, we show a novel role for intracellular Gal-9 in regulating the actions of its receptor, Tim-3, and immune responses during M/M_Ø_ activation. We demonstrate that Tim-3 and IL-12/IL-23 gene transcriptions are regulated by altered levels of Gal-9 expression in monocytes through synergizing with TLR signaling. Additionally, TLR activation promotes Gal-9/Tim-3 *cis* association within the same M/M_Ø_ to differently regulate IL-12/IL-23 expressions via STAT-3 phosphorylation. These results reveal a compartment-dependent regulatory effect of ligand (Gal-9) on its receptor (Tim-3) activities and inflammatory responses in M/M_Ø_ via TLR pathways.

## Results

### Differential regulation of Tim-3 and IL-12/IL-23 gene expressions by enhanced or silenced Gal-9 expression in THP-1 monocytic cells

**Figure 1:**
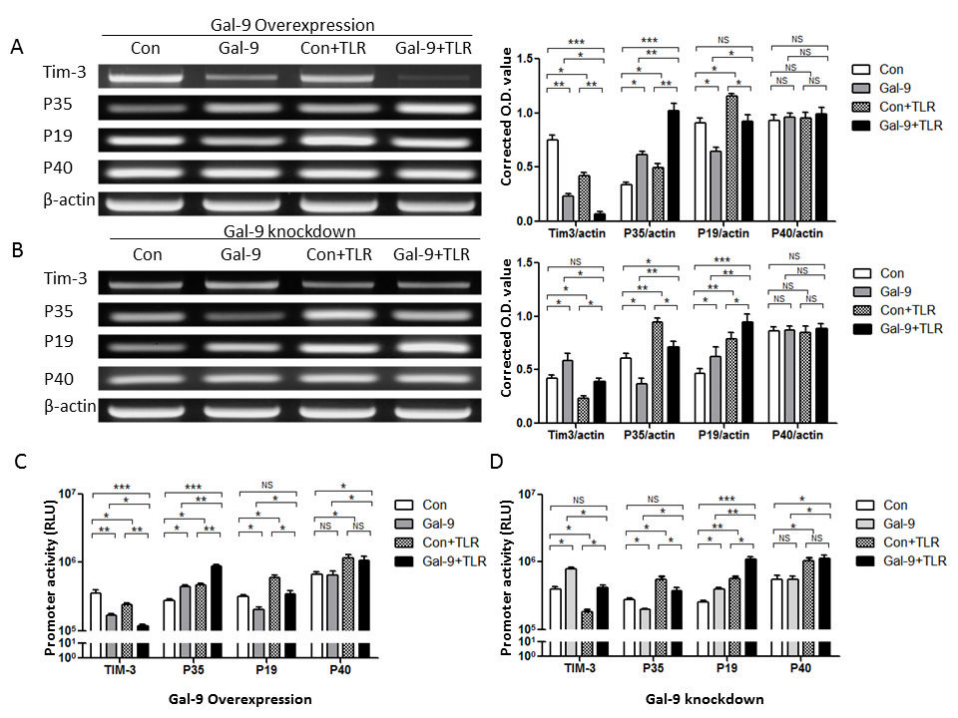
Tim-3 and IL-12/IL-23 mRNA expression in THP-1 cells with altered Gal-9 expressions with or without TLR stimulations. A) RT-PCR detection of Tim-3, IL-12p35, IL-23p19, and IL-12/IL-23p40 mRNAs in THP-1 cells transfected with Gal-9 plasmid in the absence or presence of TLR stimulations. THP-1 cells were transfected with either pBKCMV3-Gal-9 plasmid (Gal-9) or pBKCMV3 empty vector (Con) for 24 h, stimulated with or without LPS and R848 for 6 h, followed by RT-PCR measuring Tim-3, IL-12p35, IL-23p19, IL-12/IL-23p40 mRNA expressions. β-actin served as loading control to normalize target gene levels. Data are shown as representative imaging (left) and mean ± SE of corrected optimal densitometry (O.D) values from three independent experiments (right). *P<0.05, **P<0.01, ***P<0.001, NS = no significance, analyzed by multiple comparisons testing/least significant difference on the ANOVA Prism software. B) RT-PCR detection of Tim-3, IL-12p35, IL-23p19, and IL-12/IL-23p40 mRNAs in THP-1 cells transfected with Gal-9 siRNAs in the absence or presence of TLR stimulations. THP-1 cells were transfected with either Gal-9 or control siRNAs for 48 h, stimulated with or without LPS/R848 for 6 h, followed by RT-PCR measuring Tim-3, IL-12p35, IL-23p19, IL-12/IL-23p40 mRNA expressions. β-actin served as loading control. Data are shown as representative imaging (left) and mean ± SE of corrected optimal densitometry (O.D) values from three independent experiments (right). *P<0.05, **P<0.01, ***P<0.001, NS = no significance, analyzed by multiple comparisons testing/least significant difference on the ANOVA Prism software. C) Enhanced Gal-9 expression and TLR stimulation on Tim-3, IL-12, IL-23 promoter activities. pBKCMV3-Gal-9 or control vector was transiently transfected into THP-1 cells, along with either Tim-3, or IL-12p35, or IL-23p19, or IL-12/IL-23p40 promoter luciferase reporter vectors. 24 h after transfection, the cells were stimulated with or without LPS/R848 for 6 h, followed by luciferase assays for reporter gene transcriptional activities as described in Methods. Data are shown as mean ± SE of triplicate samples for relative luciferase units (RLU). *P<0.05, **P<0.01, ***P<0.001, NS = no significance, analyzed by multiple comparisons testing/least significant difference on the ANOVA Prism software. D) Silenced Gal-9 expression and TLR stimulation on Tim-3, IL-12, IL-23 promoter activities. Gal-9 or control siRNAs was transiently transfected into THP-1 cells, along with either Tim-3, or IL-12p35, or IL-23p19, or IL-12/IL-23p40 promoter reporter vectors. 48 h after transfections, the cells were stimulated with or without LPS/R848 for 6 h, followed by luciferase assays. Data are shown as mean ± SE of triplicate samples for relative luciferase units (RLU). *P<0.05, **P<0.01, ***P<0.001, NS = no significance, analyzed by multiple comparisons testing/least significant difference on the ANOVA Prism software.

While the Tim-3/Gal-9 interaction has been shown to be a critical mechanism for T cell exhaustion [17–25], its role in regulating innate immunity is controversial [10–16]. We have previously shown that Tim-3 expressed on the surface of M/M_Ø_ inhibits IL-12 production through its binding to extracellular Gal-9, either soluble or presented by other cells (a *trans* association) [27–30]. Since Gal-9 is also expressed in M/M_Ø_, whether intracellular Gal-9 may regulate the function of its receptor (Tim-3) and inflammatory responses (IL-12/IL-23) within the same cells is studied here. To this end, we first examined the Tim-3 and IL-12/IL-23 mRNA expressions in THP-1 cells transfected with either a Gal-9 expressing plasmid or Gal-9 silencing siRNA. As shown in Figure 1A left panel, over-expression of Gal-9 in THP-1 cells results in lower levels of Tim-3 and IL-23p19, higher levels of IL-12p35, but no significant change of IL-12/IL-23p40 mRNA expressions, compared with those transfected with a control plasmid. In contrast, siRNA knockdown of Gal-9 activates Tim-3 and IL-23p19, inhibits IL-12p35, but does not affect IL-12/IL-23p40 mRNA levels (Figure 1B left panel). These results indicate that intracellular Gal-9 activates IL-12 and inhibits Tim-3 and IL-23 gene expressions.

To confirm these results, the effects of intracellular Gal-9 on Tim-3 and IL-12/IL-23 gene promoter activities were examined by luciferase assay. Briefly, Gal-9 plasmid was co-transfected into THP-1 cells along with Tim-3, IL-12, or IL-23 promoter luciferase reporter genes. As shown in Figure 1C left panel, over-expression of Gal-9 results in a similar inhibition of Tim-3 and IL-23p19, an increase of IL-12p35, but no significant change in IL-12/IL-23p40 promoter activities. In contrast, silencing Gal-9 expression enhances Tim-3 and IL-23p19, diminishes IL-12p35, but does not alter IL-12/IL-23p40 promoter activities, when compared with their controls (Figure 1D left panel). These results suggest that intracellular Gal-9 functions as a modulator of Tim-3 and inflammatory cytokine IL-12/IL-23 gene transcriptions.

### Intracellular Gal-9 alters Tim-3 and IL-12/IL-23 gene expressions through synergizing with TLR pathways

It is well-established that the inflammatory response is initiated as M/M_Ф_ activation and cytokine expression through TLR signaling pathways [1]. Of note, lipopolysaccharide (LPS) enhances Gal-9 expression [32]. To determine whether intracellular Gal-9 alters Tim-3 and IL-12/IL-23 gene expressions through TLR pathways, THP-1 cells were transfected with Gal-9 expressing plasmid followed by stimulation with TLR4 ligand-LPS and TLR7/8 ligand-R848, which are essential for IL-12/IL-23 expressions [33,34]. Compared with transfected THP-1 cells without TLR stimulation, LPS/R848 treatment significantly reduces Tim-3, but boosts IL-12p35 and IL-23p19 mRNA expressions (Figure 1A right panel). Notably, Gal-9-transfected THP-1 cells with TLR stimulation exhibit significantly decreased Tim-3 and increased IL-12p35 mRNA expressions compared to THP-1 cells transfected with control plasmid, particularly to those without TLR stimulation. Perhaps due to the opposing effects of Gal-9 and TLR stimulation on IL-23p19 mRNA expression, IL-23p19 is still significantly reduced in Gal-9-transfected cells versus mock transfection following TLR stimulation, but its level has no significant change when compared with mock transfection without TLR stimulation.

Conversely, we also assayed whether intracellular Gal-9 alters Tim-3 and IL-12/IL-23 gene expressions through TLR pathways by knockdown of Gal-9 expression in THP-1 cells with LPS/R848 stimulation. As shown in Figure 1B right panel, TLR stimulation reduces Tim-3, activates IL-12p35 and IL-23p19, but does not changes IL-12/IL-23p40 mRNA expressions in THP-1 cells transfected with Gal-9 or control siRNAs; however, silencing Gal-9 expression in the setting of TLR stimulation still activates Tim-3 and IL-23p19, inhibits IL-12p35, and does not alter IL-12/IL-23p40 mRNA expressions compared to mock transfection with TLR stimulation; this leads to a minor alteration of Tim-3, modest activation of IL-12, and significant increase of IL-23 mRNA expressions, compared to mock-transfected THP-1 cells without TLR stimulation.

To confirm these results, we reexamined the effects of intracellular Gal-9 on Tim-3 and IL-12/IL-23 transcriptional activities by co-transfecting THP-1 cells with a Gal-9 plasmid along with Tim-3, IL-12, or IL-23 promoter genes followed by TLR stimulation and luciferase activity assay. As shown in Figure 1C right panel, TLR stimulation reduces Tim-3, but activates IL-12/IL-23 promoter activities in transfected cells. Again, enhanced Gal-9 expression by TLR stimulation inhibits Tim-3 and IL-23p19, activates IL-12p35, and does not alter IL-12/IL-23p40 promoter activities compared to mock transfection with TLR stimulation; leading to a very significant inhibition of Tim-3, and very significant activation of IL-12, but no significant change of IL-23 promoter activities, when compared to mock-transfected THP-1 cells without TLR stimulation. As shown in Figure 1D right panel, however, diminished Gal-9 expression in the setting of TLR stimulation activates Tim-3 and IL-23p19 but inhibits IL-12p35 promoter activities compared to mock transfection with TLR stimulation; leading to little change of Tim-3 and IL-12, but very significant increase of IL-23 promoter activities, when compared to THP-1 cells treated with control plasmid without TLR stimulation. These results indicate that intracellular Gal-9 modulates its receptor Tim-3 and cytokine IL-12/IL-23 transcriptional activities through synergizing with TLR pathways.

### Gal-9 differentially regulates IL-12 and IL-23 gene transcriptions through STAT-3 signaling

**Figure 2:**
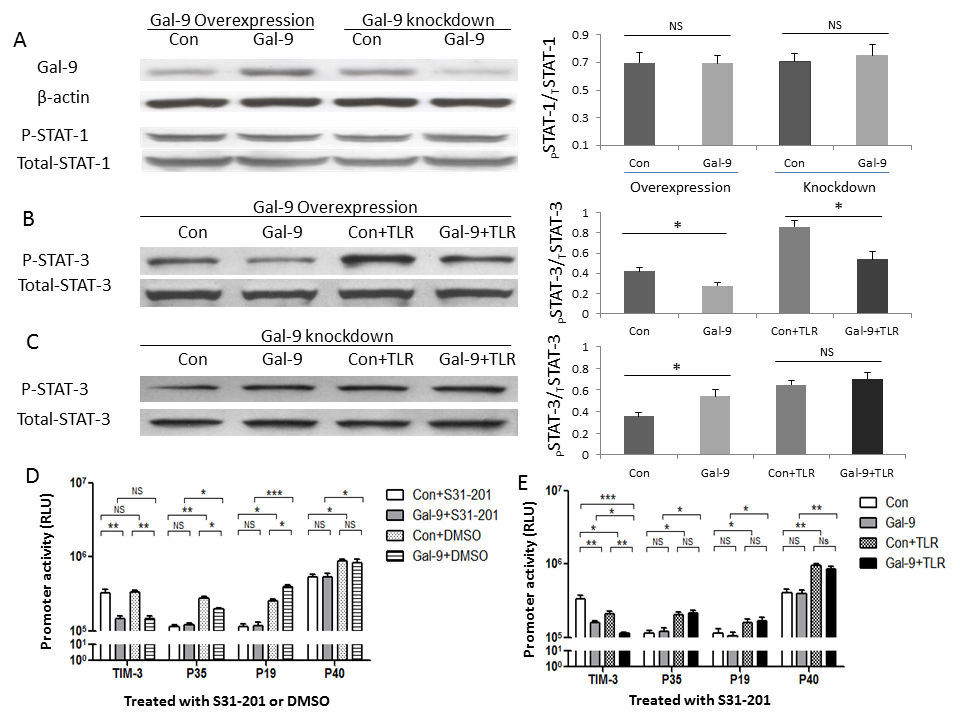
Gal-9 differentially regulates IL-12 and IL-23 gene transcriptions in THP-1 cells through STAT-3 signaling. A) Western blot detection of Gal-9 and phosphorylation of STAT-1 proteins in THP-1 cells transfected with Gal-9 plasmid or siRNA. THP-1 cells were transfected with either pBKCMV3-Gal-9 plasmid (Gal-9) or pBKCMV3 empty vector (Con), or Gal-9 silencing siRNA or control siRNA. After 24~48 h transfection, the cells were subjected to Western blot analysis of Gal-9 and pSTAT-1 proteins. β-actin or total STAT-1 served as loading control to normalize target gene levels. Data are shown as representative imaging (left) and corrected optimal densitometry (O.D.) values from three independent experiments for STAT-1 (right). NS = no significance. B) Western blot detection of pSTAT-3 protein in THP-1 cells transfected with Gal-9 plasmid in the presence or absence of TLR stimulations. THP-1 cells were transfected with either Gal-9 or Control plasmid for 24 h, stimulated with or without LPS/R848 for 6 h, followed by Western blot analysis of pSTAT-3 protein. Total STAT-3 served as loading control to normalize target gene levels. Representative imaging and corrected O.D. values from three independent experiments are shown. *P<0.05. C) Western blot detection of pSTAT-3 protein in THP-1 cells transfected with Gal-9 siRNAs with or without TLR stimulations. THP-1 cells were transfected with either Gal-9 or control siRNAs for 48 h, stimulated with or without LPS/R848 for 6 h, followed by Western blot analysis of pSTAT-3 protein. Total STAT-3 served as loading control. Representative results and corrected O.D. values from three experiments are shown. *P<0.05, NS = no significance. D) Luciferase assay for Tim-3, IL-12, IL-23 promoter activities in Gal-9-transfected THP-1 cells in the presence of STAT-3 inhibitor. pBKCMV3-Gal-9 or control plasmid was transfected into THP-1 cells, along with Tim-3, IL-12p35, IL-23p19, or IL-12/IL-23p40 promoter reporter vectors, in the presence of STAT3-specific inhibitor or DMSO for 24 h, followed by luciferase assays. Data are mean ± SE of triplicate samples for relative luciferase units (RLU). *P<0.05, **P<0.01, ***P<0.001, NS = no significance. E) Intracellular Gal-9 and TLR stimulation on Tim-3, IL-12, IL-23 promoter activities in THP-1 cells treated with STAT-3 inhibitor and TLR stimulation. pBKCMV3-Gal-9 or control vector was transfected into THP-1 cells, along with Tim-3, IL-12p35, IL-23p19, or IL-12/IL-23p40 promoter reporter vectors, in the presence of STAT3-specific inhibitor for 24 h. The cells were stimulated without or with LPS/R848 for 6 h, followed by luciferase assays as described in Methods. Data are mean ± SE of triplicate samples for relative luciferase units (RLU). *P<0.05, **P<0.01, ***P<0.001, NS = no significance.

After binding pathogens, TLR signaling leads to crosslinking of adapter proteins and activation of several protein kinase pathways, including JAK/STAT [1]. To determine whether intracellular Gal-9 differentially regulates IL-12 and IL-23 gene transcriptions in M/M_Ф_ through JAK/STAT pathways, THP-1 cells transfected with a Gal-9 expressing plasmid or silencing siRNA were subjected to Western blot analysis of phosphorylations of STAT-1 (Tyr701) and STAT-3 (Tyr705). Indeed, THP-1 cells transfected with a Gal-9 expressing plasmid exhibit increased Gal-9 expression whereas those transfected with Gal-9 silencing siRNA show decreased Gal-9 protein compared to those transfected with controls (Figure 2A). Significant increases of Gal-9 expression is primarily found intracellularly when detected by flow cytometry, rather than extracellularly when assayed by ELISA, especially in the setting of TLR stimulation--a condition that favors cis rather than trans association (Figure S1 A and B). However, phosphorylation of STAT-1 is similar in Gal-9 transfected or silenced cells. Notably, over-expression of Gal-9 in THP-1 cells significantly inhibits the phosphorylation of STAT-3 compared to those transfected with the control plasmid (Figure 2B). Although TLR stimulation activates the phosphorylation of STAT-3 protein, enhanced Gal-9 expression in the setting of TLR activation still inhibits STAT-3 phosphorylation compared to those treated with the control plasmid plus TLR ligands.

We also assayed whether intracellular Gal-9 alters STAT-3 phosphorylation in the cascade of TLR signaling by knockdown of Gal-9 expression in THP-1 cells transfected with siRNA at 48 h, stimulation with LPS/R848 for 6 h, and then evaluation of STAT-3 activation by Western blot. As shown in Figure 2C, diminished Gal-9 expression activates STAT-3 phosphorylation compared with the control. Again, TLR stimulation increases STAT-3 phosphorylation; however, silencing Gal-9 gene expression further enhances STAT-3 phosphorylation in THP-1 cells treated with Gal-9 siRNAs and TLR stimulation.

To further evaluate the role of STAT-3 in Gal-9-mediated regulation of Tim-3 and IL-12/IL-23 transcriptional activities, we co-transfected THP-1 cells with Gal-9 plasmid along with Tim-3, IL-12, or IL-23 promoter genes in the presence of a specific STAT-3 inhibitor (S31-201) or DMSO control for 24 h, stimulated the cells with or without LPS/R848 for 6 h, followed by luciferase assay for their promoter activities. As shown in Figure 2D, THP-1 cells treated with a STAT-3 inhibitor exhibit diminished IL-12p35, IL-23p19, and IL-12/IL-23p40 promoter activities compared with those exposed to the DMSO control. This is consistent with our previous finding that IL-12 and IL-23 cytokine productions were significantly diminished in M/M_Φ_ treated with a STAT-3 inhibitor and assayed by flow cytometric analysis [29]. Notably, Gal-9 transfection does not affect IL-12p35 and IL-23p19 or IL-12/IL-23p40 transcriptional activities in the presence of the STAT-3 inhibitor, but does alter IL-12p35 and IL-23p19 transcriptional activities in the presence of DMSO, suggesting that intracellular Gal-9 alters IL-12 and IL-23 gene transcriptions via STAT-3 signaling. Interestingly, Gal-9 transfection still inhibits Tim-3 promoter activity to the same level regardless of the presence or absence of STAT-3 inhibitor and DMSO (Figures 1C and 2D), indicating that Gal-9 likely affects Tim-3 gene transcription through pathways other than STAT-3. As shown in Figure 2E, TLR stimulation reduces Tim-3 and activates IL-12p35, IL-23p19, and IL-12/IL-23p40 transcriptional activities in THP-1 cells transfected with Gal-9 or control plasmids in the presence of the STAT-3 inhibitor. However, the baseline levels of IL-12p35 and IL-23p19 promoter activities are greatly diminished in the presence of a STAT-3 inhibitor when compared to those stimulated with or without LPS in the absence of STAT-3 inhibitor (Figures 1C and 2E); the IL-12/IL-23p40 promoter still responds well to LPS stimulation, indicating that--in addition to STAT-3 signaling--LPS/R848 must also trigger IL-12 and IL-23 gene transcriptions through other pathways. Nevertheless, enhanced Gal-9 expression does not alter IL-12 and IL-23 gene transcriptions in the presence of TLR stimulation and the STAT-3 inhibitor, confirming the critical role of STAT-3 in Gal-9-induced differential regulation of IL-12p35 and IL-23p19 expressions. These results indicate that intracellular Gal-9 modulates inflammatory cytokine IL-12/IL-23 gene transcriptions through STAT-3 signaling.

### TLR stimulation enhances Gal-9/Tim-3 cis association and activates monocyte IL-12/IL-23 expressions

**Figure 3:**
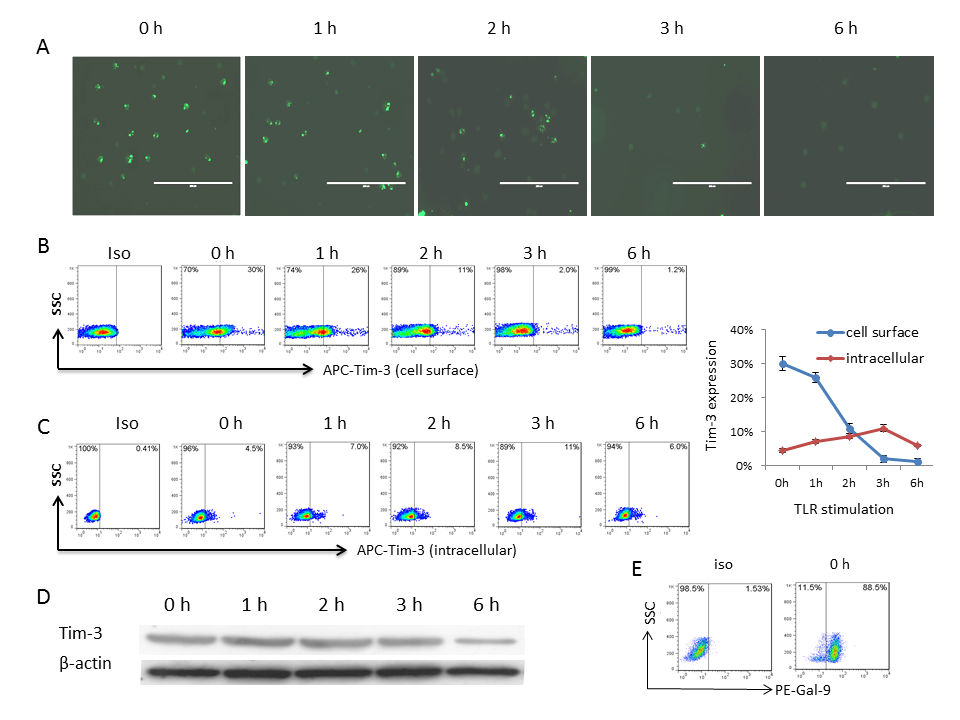
Tim-3 internalization from cell surface into cytoplasm of M/M_Φ_ with bounty of Gal-9 following TLR stimulation. A) The kinetic presentation of Tim-3 on the surface of M/M_Ø_ following TLR stimulation analyzed by immunofluorescent microscopy. Tim-3 on the surface of live M/M_Ø_ was labeled with a primary Tim-3 antibody (Santa Cruz); the cells were stimulated with LPS/R848 for 0, 1, 2, 3, 6 h, followed by staining with a fluorescence-conjugated secondary antibody and analysis by AMG fluorescence phase microscope. Magnification 20x for all panels and scale bar = 100 µm. B) The presentation of Tim-3 on the surface of M/M_Ø_ following TLR stimulation analyzed by flow cytometry. M/M_Ø_ were stimulated with LPS/R848 for the same period of time (0, 1, 2, 3, 6 h), immunostained for Tim-3 cell surface expression using a different source of antibody (eBioscience), and analyzed by flow cytometry as described in the Methods. Isotype-matched control antibodies (eBioscience) and fluorescence minus one (FMO) controls were used to determine background levels of staining and adjust multicolor compensation as gating strategy. Representative dot plots with percentages of cells in the gated area are shown. C) The presentation of Tim-3 inside the cytoplasm of M/M_Ø_ following TLR stimulation analyzed by flow cytometry. First, M/M_Ø_ were incubated with 10 µg/ml of un-conjugated mAb (F38-2E2, Biolegend) to saturate surface Tim-3 epitopes; after TLR stimulation for the same period of time and fixation/permeabilization procedures, staining of intracytoplasmatic Tim-3 epitopes was performed using the same clone of conjugated Tim-3 mAb (F38-2E2), followed by flow cytometric analysis. Representative dot plots with percentages of cells gated based on the same strategy are shown on the left. The percentage of Tim-3 presentation (mean ± SD) on the surface and intracellular of M/M_Ø_ following TLR stimulation analyzed by flow cytometry from 3 independent experiments is shown on the right. D) The amount of Tim-3 in M/M_Ø_ following TLR stimulation analyzed by Western blot. Purified M/M_Ø_ were stimulated with LPS/R848 for the same period of times, and total Tim-3 protein expression was detected by Western blot as described in the Methods. E) Representative dot plots for intracellular Gal-9 detection in purified resting human M/M_Ø_ by flow cytometry.

We have previously shown that Tim-3 is constitutively expressed on resting M/M_Ø_ and functions as a cap, declining rapidly upon TLR stimulation and allowing IL-12 and IL-23 expression in activated cells [27–30]. To further evaluate the kinetic behavior of Tim-3 in M/M_Ø_ following TLR stimulation, we labeled Tim-3 on the surface of live M/M_Ø_ with primary antibody (Santa Cruz, N-16), and then stimulated with or without LPS/R848 for various times, followed by fluorescent-conjugated secondary antibody staining and analysis with AMG fluorescence phase microscopy. As shown in Figure 3A, Tim-3 is widely expressed on the surface of non-stimulated M/M_Ø_ (0 h), rapidly declines during the first 1~3 h, and almost disappears at 6 h on the cell surface following TLR stimulation; whereas Tim-3 expression remains unchanged on the cell surface without TLR stimulation (data not shown). To exclude off-target effects of the TLR ligands, we also examined Tim-3 surface expression on cells stimulated with LPS with or without anti-human CD284 antibody (TLR4 blocking antibody, 10 µg/ml, clone HTA 125, eBioscice). Indeed, by blocking LPS interactions with TLR4, the reduction of Tim-3 cell surface expression is significantly inhibited (Figure S1 C). These results are confirmed by incubating purified M/M_Ø_ with and without LPS/R848 for the same period of time (0, 1, 2, 3, and 6 h), immunostaining for Tim-3 cell surface expression with a different source of antibody (eBioscience, APC-F38-2E2) and analyzing by flow cytometry (Figure 3B).

Since Gal-9 is highly expressed intracellularly in M/M_Ø_, we queried whether Tim-3 and Gal-9 expressed within the same M/M_Ø_ might undergo a receptor/ligand *cis* association, and what would result from such an interaction. Of note, TLR stimulation inhibits Tim-3 mRNA expression (Figure 1) but activates Gal-9 translocation and gene expression [31,32]. Given the fact that gene transcription/translation takes several hours, while Tim-3 cell surface decline occurs rapidly, upon TLR stimulation (Figure 3A and B), thus a *cis* association of Tim-3/Gal-9 might be even more important in the early phase of TLR stimulation, which may activate Gal-9 and/or Tim-3 phosphorylation or confirmation changes due to their interactions. Indeed, it has been reported that a highly conserved SH2 domain in the intracellular tail of Tim-3 is tyrosine (Y265) phosphorylated upon stimulation by its ligand, Gal-9 [35]. In order to assay for the presence of an intracellular Gal-9/Tim-3 *cis* association, we first examined the possibility that cell surface Tim-3 might be internalized into the M/M_Ø_ upon TLR stimulation. To this end, a two-step labeling of TLR-stimulated M/M_Ø_ was performed and analyzed by flow cytometry. First, M/M_Ø_ were incubated with 10 µg/ml of un-conjugated mAb (F38-2E2, Biolegend) to saturate surface Tim-3 epitopes (Tim-3 cell surface staining negative); after TLR stimulation and fixation/permeabilization procedures, staining of intracytoplasmatic Tim-3 epitopes was performed using the same clone of FITC-conjugated Tim-3 mAb (F38-2E2). As shown in Figure 3C dot plots and summary data, Tim-3 expression, while rapidly declining at 1~3 h from the cell surface, slowly rises intracellularly during 1~3 h, and then drops at 6 h following TLR stimulation. However, no significant change of total Tim-3 protein is detected by Western until 6 h, by which time a decline of Tim-3 level is detected in M/M_Ø_ following TLR stimulation (Figure 3D). These results suggest the possibility of early Tim-3 translocation from cell surface into cytoplasm in 1~3 h, then degradation through proteolysis by 6 h, after TLR stimulation.

**Figure 4:**
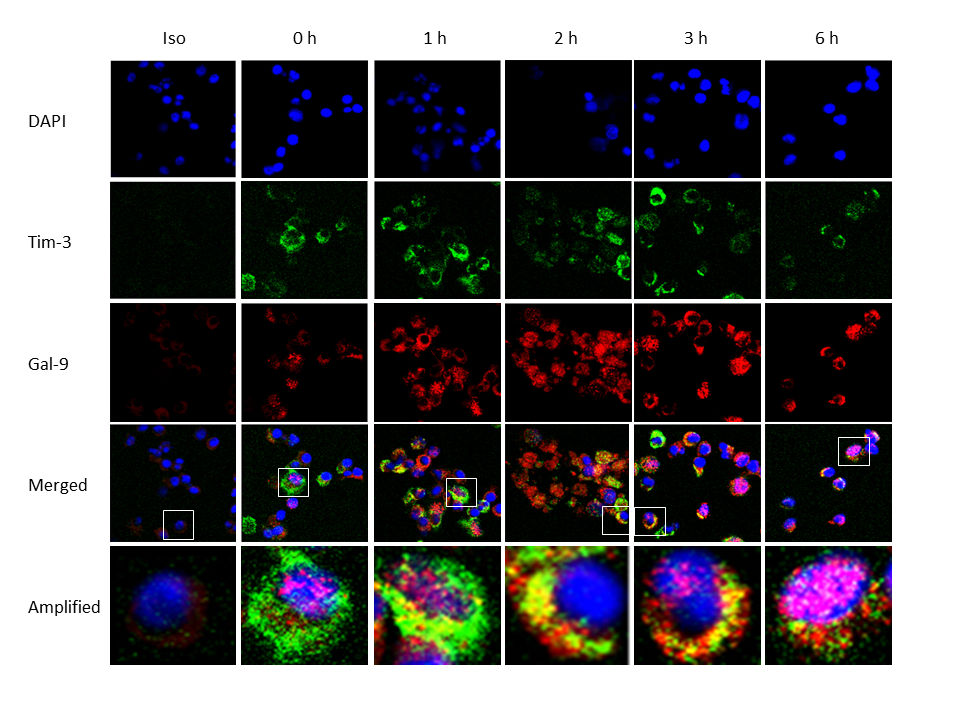
TLR stimulation enhances Tim-3/Gal-9 *cis* association that is co-localized intracellular of M/M_Ø_. Detection of Tim-3 and Gal-9 localization/co-localization in resting and TLR-stimulated M/M_Ø_ by confocal microscopy. Purified M/M_Ø_ were stimulated with LPS/R848 for 0, 1, 2, 3, 6 h; following fixation/permeabilization, intracytoplasmic Tim-3 and Gal-9 staining as well as DAPI nuclear staining (blue), isotype control staining, and their imaging merges observed by confocal microscopy as described in the Methods. Magnification 100x and 1.4 oil DIC (total of 140x) for all panels. A typical cell with Tim-3 (green) expression, Gal-9 (red) expression, and imaging merge as evidence of their co-localization (yellow) in M/M_Ø_ at each time point is shown (denoted in the square) and further amplified below.

**Figure 5:**
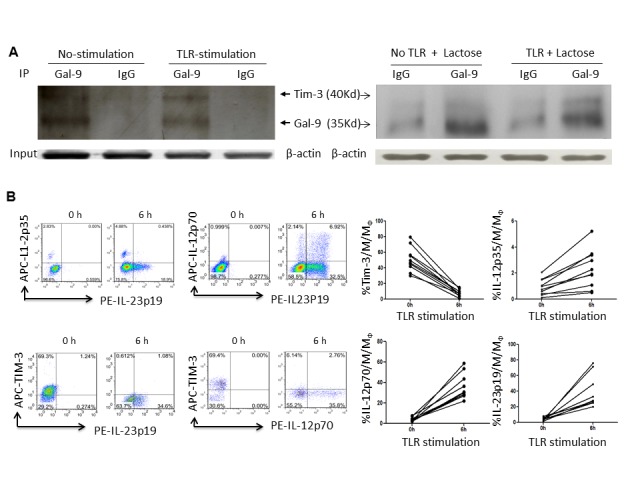
Activation of IL-12/IL-23 expressions by M/M_Ø_ following TLR stimulation and Tim-3/Gal-9 *cis* association. A) Detection of Tim-3/Gal-9 physical association in un-stimulated and TLR-stimulated M/M_Ø_ by immunoprecipitation. The purified M/M_Ø_ were stimulated with or without LPS/R848 for 6 h; the procedure for co-immunoprecipitation of Tim-3 (40 Kd) and Gal-9 (35 Kd) was described in the Methods. Samples were pulled down by anti-Gal-9 or IgG antibodies and protein A/G PLUS-agarose; then probed with anti-Tim-3 and HRP-secondary antibody. β-actin was used to probe cell lysates for equal protein inputs. Repeated co-ip experiment by M/M_Ø_ in the presence of α-lactose in the cell lysates to inhibit random Gal-9 bound of Tim-3 are shown in the right panel. B) Activation of IL-12/IL-23 expressions by M/M_Ø_ following TLR stimulation. Purified M/M_Ø_ were cultured in the presence or absence of LPS/R848 for 6 h. Tim-3 cell surface expression and intracellular IL-12p35, IL-12p70, IL-23p19 productions were analyzed by flow cytometry as described in the Methods. Representative dot plots of the relationship between IL-12p35 and IL-23p19, IL-12p70 and IL-23p19, Tim-3 and IL-23p19, Tim-3 and IL-12p70, and summary data derived from multiple subjects were shown. Each line-linked symbol represents one subject’s M/M_Ø_ before and after TLR stimulation.

Constitutively and primarily, Tim-3 is expressed on the cell surface, while Gal-9 is expressed in the cytoplasm, of resting M/M_Ø_ (Figure 3A–E), and thus their interactions within the same cell might only occur upon TLR stimulation, a trigger of Tim-3/Gal-9 *cis* associations. To test this possibility, we further evaluated Tim-3 and Gal-9 localization/co-localization in resting and TLR-stimulated M/M_Ø_ by intracellular immunofluorescent staining for Tim-3 (green), Gal-9 (red), and DAPI nuclei staining (blue). Overlaid imaging was observed with fluorescence microscopy (Figure S2) as well as confocal microscopy (Figure 4), which clearly shows Tim-3 surface (green) and Gal-9 intracellular (red) localization in resting M/M_Ø_ (0 h) and their co-localization intracellularly (yellow) upon TLR stimulation. More specifically, yellow pixels in a red-green merged image was barely observed at resting M/M_Ø_, but clearly showed up at early phase of TLR stimulation. We also detected Tim-3/Gal-9 physical association in the resting and TLR-stimulated M/M_Ø_ by immunoprecipatation (ip), with result in Figure 5A showing that Tim-3 can be pulled-down by Gal-9 in the TLR-stimulated M/M_Ø_ more than those unstimulated cells. To exclude the possibility of non-specific association of Gal-9 with Tim-3 after TLR stimulation, repeated co-ip experiments were carried out by TLR-stimulated M/M_Ø_ in the presence of α-lactose, a competitive substrate that inhibits random binding of galectins to their receptors [19]. As shown in Figure 5A right panel, a specific band for Tim-3 (40 Kd) and Gal-9 (35 Kd) proteins were detected by co-ip in TLR-stimulated M/M_Ø_, but more Gal-9 and less Tim-3 is pulled-down in unstimulated cells, indicating Tim-3 *cis* association with Gal-9 in M/M_Ø_ following TLR stimulation.

To examine the consequences of the Tim-3/Gal-9 *cis* association following TLR stimulation, we measured IL-12 and IL-23 cytokine productions in M/M_Ø_ with or without LPS/R848 stimulation. As shown in the representative dot plots and summary data in Figure 5B, IL-12 and IL-23 are barely detectable in unstimulated M/M_Ø_ that present high levels of Tim-3 surface expression. After 6 h TLR stimulation, Tim-3 declines from the cell surface; resulting in IL-12/IL-23 expressions, primarily by Tim-3 negative M/M_Ø_, suggesting that *cis* association of Tim-3/Gal-9 in M/M_Ø_ might be an early mechanism for inflammatory responses.

## Discussion

Human M/M_Ø_ are instrumental in mediating innate to adaptive immunity by secreting inflammatory cytokines that are tightly controlled by a wide variety of stimulatory and inhibitory receptors. Among them, TLR and Tim-3 are fundamental in regulating cell activation and preventing cell over-activation, and thus auto-immunity or immune-mediated injury. It is generally believed that these receptors regulate M/M_Ø_ functions by binding extracellular ligand, either soluble or presented by other cells, via a *trans* association. Little is known regarding whether and how a ligand may regulate its receptor simultaneously expressed within the same cells. This study provides pilot evidence that intracellular expression of ligand (Gal-9) can regulate receptor (Tim-3) activities and M/M_Ø_ function by at least two distinct mechanisms: i) modulation of the receptor and inflammatory cytokine gene transcription; and ii) alteration of the existing receptor translocation in M/M_Ø_ via *cis* association. Moreover, it appears that intracellular Gal-9 exerts these regulatory effects by synergizing with TLR pathways, i.e., following binding of pathogen, TLR signaling facilitates Gal-9/Tim-3 *cis* association in the early phase and subsequently inhibits Tim-3 gene transcription, resulting in down-regulation of Tim-3 cell surface presentation and differential regulation of IL-12/IL-23 productions_._

A novel finding of this study is demonstration of a ligand (Gal-9) compartment-dependent regulatory effect on its receptor (Tim-3) activities and inflammatory responses in M/M_Ø_ via TLR pathways. We have previously shown that an up-regulation of extracellular Gal-9 presented by HCV-infected hepatocytes interacting with Tim-3 highly expressed on M/M_Ø_ (an example of ligand/receptor *trans* association in the setting of pathogen infection) leads to decreased IL-12 and increased IL-23 productions by inhibiting STAT-1 and/or activating STAT-3 phosphorylation [29,30]. Here we show that intracellular Gal-9 can *cis* associate with Tim-3 within the same M/M_Ø_ and modulate its receptor as well as inflammatory cytokine gene transcription, resulting in high IL-12 and low IL-23 expressions; this effect can be amplified by TLR stimulation. At this point, we have not determined the sub-cellular compartment(s) in which this *cis* association occurs. Because internalization of receptors is generally driven by clathrin-dependent vesicle transport in which extracellular aspects of the receptor are insulated from cytoplasmic proteins, how the receptor binding sites access to ligand within the cell is as yet unclear. However, given the fact that TLR stimulation induces cell surface Gal-9 expression (unpublished data), it is feasible that *cis* association occurs on the cell surface followed by internalization. The issue of sub-cellular localization of this interaction is currently being addressed in our laboratory.

This phenomenon is similar to what has been observed in natural killer (NK) cells. Killer cell immunoglobulin-like receptors (KIR) prevent auto-aggression by blocking NK cell response to healthy autologous cells through binding human leukocyte antigen (HLA)-I presented by target cells in *trans*. However, the simultaneous expression of both HLA-I and KIRs on the same cell results in a receptor-ligand interaction within the cell itself in *cis*. What results is a remarkable reduction of KIR3DL1 surface expression when the cognate ligand is present on the same human NK cell, leading to KIA/HLA-I *cis* association that is able to influence NK cell cytotoxic function and licensing [36,37]. Moreover, *cis* association between mouse MHC-I (H-2d) and Ly49 (the murine counterpart of KIR) have been shown to both reduce surface expression of Ly49 and to allow NK cell licensing in mice [38,39]. Thus, the lower surface staining of Ly49 is due at least in part to sequestration and translocation when this molecule associates with MHC-I in *cis*. Similarly, here we show Tim-3/Gal-9 *cis* association within individual M/M_Ø_ restricts the number of Tim-3 receptors available for binding of Gal-9 on other cells in *trans* and reduces M/M_Ø_ inhibition and/or promotes cell activation. Consequently, by lowering the threshold at which M/M_Ø_ activation exceeds inhibition, *cis* interaction may facilitate an optimal immune response to pathogen-infected cells.

In addition to engaging with Tim-3 to regulate cell functions, Gal-9 also appears to modulate its receptor and inflammatory cytokine gene transcriptions through STAT-3 signaling. To date, there exist few reports describing the intracellular function of galectins on gene regulation. For example, phosphorylation of galectin-3 contributes to malignant transformation of human epithelial cells via modulation of a unique set of genes [40]; whereas galactin-4 is involved in p27-mediated activation of the myelin basic protein promoter via its physical association with NF-IL6 [41]. Gal-9 is reported to be an LPS-responsive factor in that following LPS stimulation, Gal-9 rapidly translocates into nucleus, interacts with NF-IL6, and activates several inflammatory cytokine genes (IL-1α, IL-1β, and IFN-γ) in monocytes [31]. We have previously shown that an extracellular Gal-9 interaction with Tim-3 on M/M_Ф_ suppresses IL-12 and promotes IL-23 productions through inhibiting STAT-1 and/or activating STAT-3 phosphorylations during HCV infection [28–30]. The HCV-induced Tim-3/Gal-9-mediated imbalance of IL-12/IL-23 production by M/M_Ф_ during viral infection promotes T_H_17 cell as well as Foxp3^+^ Treg differentiation and development [29,30,42,43], perhaps contributing to persistent infection and autoimmune disorders. In this study, we demonstrate that intracellular Gal-9 activates IL-12 and inhibits IL-23 gene transcriptions through STAT-3 signaling. Interestingly, blocking the STAT-3 pathway abrogates Gal-9-mediated IL-12/IL-23 transcriptions, but fails to affect Tim-3 expression, suggesting a different mechanism for Tim-3 gene regulation. A recent study revealed that Tim-3 expression is controlled by T-bet [44], a Th1 transcription factor. Whether T-bet plays a role in regulating Tim-3 transcription in M/M_Ф_ is under investigation in our laboratory. Nevertheless, the demonstration that intracellular Gal-9 regulates Tim-3 and IL-12/IL-23 expressions via TLR pathways is an important conceptual advance.

**Figure 6:**
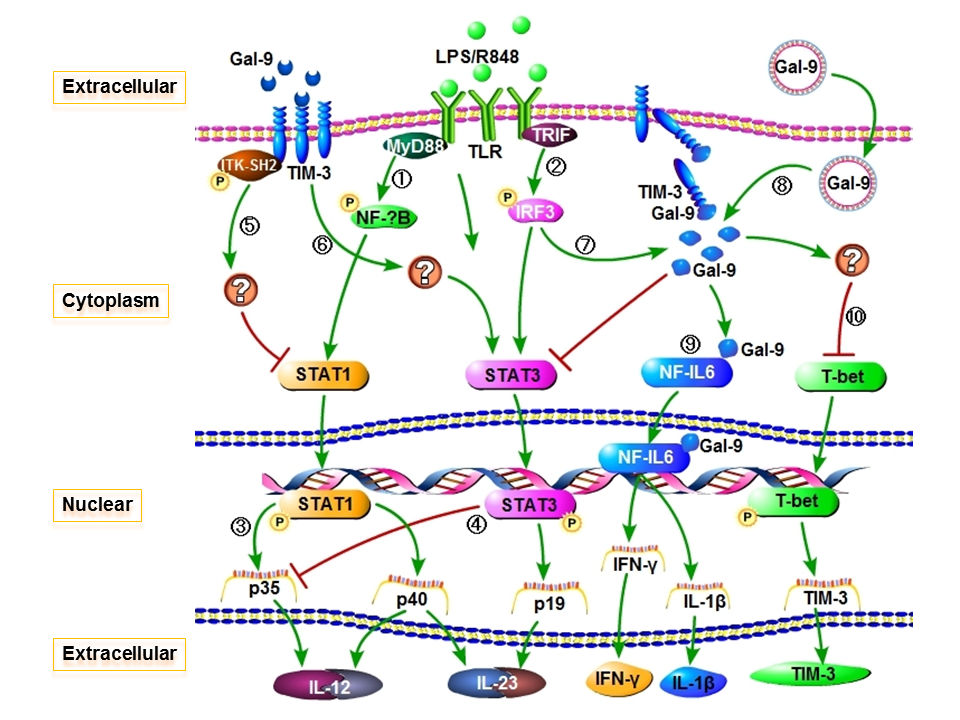
Proposed model for extracellular/intracellular Gal-9 *trans* and *cis* association with Tim-3 in regulation of monocyte inflammatory cytokine expressions through TLR signaling. After binding pathogenic products, TLR signaling involves cross-linking of adapter proteins, such as MyD88 (1) and TRIF (2), activation of NF_К_B-dependent/-independent (IRF3) pathways, and phosphorylation of multiple signaling molecules, including STAT-1 and STAT-3. Phosphorylated STAT-1 activates transcription/translation of IL-12 (3); whereas STAT-3 phosphorylation activates IL-23 but inhibits IL-12 expressions (4). In the case of chronic infections (such as HCV and HIV), pathogens may up-regulate Tim-3 expression on M/M_Φ_ and Gal-9 expression by other cells, facilitating Tim-3/Gal-9 *trans*- association and so impairing innate immune responses. More specifically, extracellular Gal-9 *trans* association with Tim-3 expressed on the surface of M/M_Φ_, triggers Tim-3-SH2 tyrosine (Y265) phosphorylation by interleukin inducible T cell kinase (ITK), delivering negative signaling to TLR-mediated STAT-1 phosphorylation (5), and positive signaling to TLR-induced STAT-3 activation (6), resulting in inhibition of IL-12 but promotion of IL-23 productions. Imbalanced IL-23/IL-12 favors the differentiation of T_H_2, TH17, and Treg cells that contribute to the development of chronic infection and autoimmune disorders. In the case of acute infection (such as self-limited HCV), TLR signaling may activate Gal-9 and Tim-3 *cis* association within the same M/M_Φ_ (7) and yield adequate inflammatory cytokine expressions. Moreover, intracellular Gal-9 expressions can modulate M/M_Φ_ Tim-3 and IL-12/IL-23 gene transcriptions (8). Upon TLR stimulation, intracellular Gal-9 rapidly translocates into nuclei, binds to NF-IL6 but not NF_К_B, and triggers IL-1β and IFN-γ transcriptions^31^ (9). Intracellular Gal-9 also inhibits STAT3 but not STAT-1 phosphorylation, leading to increased IL-12 and decreased IL-23 gene transcriptions. However, intracellular Gal-9 appears to reduce Tim-3 cell surface expression by two distinct mechanisms: *cis* association/internalization and transcriptional inhibition, through a STAT-3-independent but possibly T-bet-dependent pathway (10). Appropriate TLR signaling in innate immune cells may thus facilitate proper adaptive immune responses to successfully clear the acute infection. Based on this schematic model, therapeutic strategies manipulating Tim-3/Gal-9 *trans* and/or *cis* association might be of clinical benefit.

Based on these findings, we propose a working model, as depicted in Figure 6, for Tim-3/Gal-9 interactions in regulating M/M_Ø_ functions and inflammatory responses. This model might be plausible in understanding the role of Tim-3/Gal-9 partnership in multiple clinical scenarios, including immune tolerance, autoimmunity, antitumor and/or antiviral immune evasion. For example, during acute versus chronic phase of pathogenic infections, TLRs are activated differently by sensing and responding to pathogenic products, leading to a cascade of protein phosphorylations and multiple gene transcriptions. *Trans* association of Gal-9/Tim-3 negatively regulates TLR signaling, whereas *cis* association of Gal-9/Tim-3 positively activates this pathway. In the early phase of infections, enhancing *cis* association of Gal-9/Tim-3 ensures an appropriate TLR signaling in M/M_Ф_ that facilitate proper adaptive immune responses to successfully clear an acute infection; whereas some persistent pathogens such as HCV or HIV may enhance *trans* association of Gal-9/Tim-3 by up-regulating Gal-9 serum level and/or Tim-3 cell surface expression [45–48], and thus inhibit innate to adaptive immune responses and favor chronic infection. Therefore, understanding this mechanism in human M/M_Ф_ is fundamentally important and clinically significant, raising the possibility of manipulating the *trans* and/or *cis* association of Tim-3/Gal-9 for improving host immune responses.

## Materials and Methods

### Cell isolation & cultures

Human peripheral blood mononuclear cells (PBMCs) were isolated from the peripheral blood of healthy donors (Key Biologics LLC, Memphis, TN) by Ficoll-density centrifugation with lympho-H (Atlanta biological, Lawrence-ville, GA). CD14^+^ M/M_Ø_ were purified from PBMCs by magnetic beads with column purification according to the manufacturer’s instructions (purity > 95%; Miltenyi Biotec Inc, Auburn CA). Human THP-1 monocytic cell line was purchased from American Type Culture Collection (ATCC, Manassas, VA). The cells were cultured with RPMI 1640, containing 10% fetal bovine serum (FBS, Life Technologies, Gaithersburg, MD), 100 mg/ml penicillin-streptomycin (Thermo Scientific Logan, Utah), and 2 mM L-glutamine (Thermo Scientific, Logan, Utah) at 37° C with 5% CO_2_ atmosphere for the following experiments.

### Plasmid & siRNA Transfections

5 x 10^5^ THP-1 cells were transfected with 1.5 µg pBKCMV3-Gal-9 or control plasmid (GalPharma, Kagawa, Japan), with or without Tim-3, IL-12p35, IL-23p19, or IL-12/IL-23p40 promoter gene in pXGN-mGL bicistronic reporter vectors (Xactagen LLC, Shoreline, WA), using TransIT-TKO^®^ Transfection reagent (Mirus, Madison, WI) per manufacturer’s instructions. 50 nM Gal-9 siRNA (sc-35444) or control siRNA-A (sc-37007, Santa Cruz Biotechnology, Santa Cruz, CA) were transfected with or without mentioned promoter gene reporter vectors with the same protocol. Following 24~48 h of transfection or co-transfections, the cells were stimulated with or without 1 µg/ml of TLR 4 ligand - lipopolysaccharide (LPS, Santa Cruz) and 2.5 µg/ml of TLR 7/8 ligand - R848 (Santa Cruz) for 6 h, followed by detection of Tim-3, IL-12p35, IL-23p19, and IL-12/IL-23p40 gene transcriptions with reverse transcription-polymerase chain reaction (RT-PCR) and luciferase assay as described below.

### RT-PCR & Luciferase Assay

Total RNAs were isolated using QIAGEN Rnasy Mini Kit (QIAGEN, Valencia, CA) from THP-1 cells following transfections. 1 µg/milliliter of RNAs were reverse-transcribed (Ambion, Austin, TX) and the cDNAs were amplified by PCR (5 PRIME, Gaithsburg, MD) at conditions: 95° C 10 min followed by 95° C 45 s, 60° C 45 s, 72° C 45 s for 30~35 cycles, and then 72° C 5 min. The primers for target gene amplifications are listed in Table 1 (Integrated DNA Technologies, Coralville, IA). The amplified products were analyzed by electrophoresis on 2% agarose gels. Optimal densitometry (OD) value of the DNA products is determined by Gal-Pro Analyzer (Version 4.0, Media Cybernetics, LP).

**Table 1:** Primers for Tim-3, IL-12p35, IL-23p19, and IL-12/IL-23p40 gene amplification.

**Target genes**		**Sequence**
Tim-3	Sense	**5’-TCC AAG GAT GCT TAC CAC CAG-3’**
	Antisense	**5’-GCC AAT GTG GAT ATT TGT GTT AGA T-3’**
IL-12p35	Sense	**5’-TCC TCC TGG ACC ACC TCA GTT TG-3’**
	Antisense	**5’-TGT CTG GCC TTC TGG AGC ATG T-3’**
IL-23p19	Sense	**5’-CTC AGC AGA TTC CAA GCC TCA GTC-3’**
	Antisense	**5’-GCC TTT AGG GAC TCA GGG TTG C-3’**
IL-12/IL-23p40	Sense	**5’-GGA CCT TGG ACC AGA GCA GTG A-3’**
	Antisense	**5’-TGT GAA GCA GCA GGA GCG AAT G-3’**
β-actin	Sense	**5’-TCA CCC ACA CTG TGC CCA TCT AC-3’**
	Antisense	**5’-GAG TAC TTG GGC TCA GGA GGA GC-3’**

Gaussia luciferase assay was carried out in a 96-well format following co-transfection of THP-1 cells with Gal-9 expressing plasmid or silencing siRNA and Tim-3, IL-12p35, IL-23p19, or IL-12/IL-23p40 promoter reporter vectors. Briefly, 2 x 10^4^/20 µl transfected cells, in the presence or absence of 100 µM of STAT3-specific inhibitor S3I-201 (NSC 74859, Santa Cruz Biotechnology, CA), were incubated with 50 µl/well Assay Solution containing substrate and assay buffer at room temperature for 10 min. Luminescence (5 second integration, ~475 nm) was read by GloMax^®^-Multi Microplate Multimode reader (Promega, Madison, WI).

### Flow cytometry & Immune fluorescence

Purified human CD14^+^ M/M_Φ_ were stimulated by 1 µg/ml of LPS and 2.5 µg/ml of R848 (Santa Cruz) for 1~6 h, Brefeldin A (BioLegend, San Diego, CA) was added 6 h prior to harvest the cells forbidding cytokine secretion. Specific antibody direct conjugates for cell surface staining was carried out using Tim-3-APC (eBioscience), CD14-FITC (Miltenyi Biotec Inc, Auburn CA), followed by intracellular staining for IL-12p35-APC, IL-12p70, and IL-23p19-PE (eBioscience). The intracellular cytokine staining was carried out using Inside Stain kit (Miltenyi Biotec) per manufacturer’s instructions. Isotype-matched control antibodies (eBioscience) and fluorescence minus one (FMO) controls were used to determine background levels of staining and adjust multicolor compensation as gating strategy. The cells were analyzed on a FACS Calibur flow cytometry (BD, Franklin Lakes, NJ) and FlowJo 7.6.1 software (Ashland, OR).

Tim-3 presentation on live M/M_Φ_ was detected by immune labeling with 10 µg/ml Tim-3 polyclonal primary antibody (Santa Cruz) at 37° C for 1 h, then stimulation with 1 µg/ml LPS and 2.5 µg/ml R848 stimulation at 37° C for 1~6 h, followed by staining with 1:2000 Alexa Fluor^@^ 488-conjucated donkey anti-goat secondary antibody (Life Technology, Grand Island, NY) at room temperature for 1 h. The Tim-3 fluorescence imaging on the surface of M/M_Φ_ at 0, 1, 2, 3, 6 h after TLR stimulation was detected by AMG fluorescence phase microscopy (Bothell, WA).

To determine the possibility of Tim-3 internalization by *cis* association with intracellular Gal-9 in the setting of TLR stimulation, we have checked the ability of different monoclonal antibodies (mAb) to detect intracytoplasmatic Tim-3 and Gal-9 proteins. Several mAbs fail to detect this association, likely due to the specific epitope being located at the same binding site of Tim-3/Gal-9 ligation. A two step labeling of TLR-stimulated M/M_Ø_ was performed. First, M/M_Ø_ were incubated with 10 µg/ml of un-conjugated mAb (F38-2E2, BioLegend, San Diego, CA) to saturate surface Tim-3 epitopes, and then stimulated with LPS/R848 for 0, 1, 2, 3, 6 h; after fixation/permeabilization procedures, staining of intracytoplasmatic Tim-3 epitopes was performed using the same clone of FITC-conjugated Tim-3 mAb (F38-2E2), followed by flow cytometric analysis.

Extracellular secretion of Gal-9 in the supernatant of THP-1 cells following transfection of Gal-9 or control plasmid, with or without LPS/R848 stimulation, was assayed by ELISA (Kit provided by Dr. T. Niki from GalPharma Inc.) with procedure as described [48].

Intracellular localization and co-localization of Tim-3 and Gal-9 in M/M_Ø_ were carried out by immunofluorescent staining with the same antibodies following TLR stimulation (0, 1, 2, 3, 6 h), fixation and permeabilization. For detection of nuclei, the cells were incubated with 300 nM solution of 4',6-diamidino-2-phenylindole (DAPI) dilactate in PBS for 2 min. The imaging was observed with AMG fluorescence phase microscopy (Bothell, WA) and laser scanning confocal microscopy (LSM 510 UV META, Carl Zeiss Microscope System, Germany).

### Western blot & Immunoprecipitation

The transfected THP-1 or purified M/M_Φ_ were stimulated with or without 1 µg/ml of LPS and 2.5 µg/ml of R848 (Santa Cruz) for 1~6 h as described above, the expressions of Gal-9, Tim-3, and phosphorylated STAT-1, STAT-3 were measured by Western blot. Briefly, the cells were lysed in 1x RIPA lysis buffer (Boston BioProducts Inc, Ashland, MA) supplied with protease inhibitors/phosphorylase inhibitors (Thermo Scientific, Rockford, IL) and EDTA on ice. Cell lysates were centrifuged for 15 min at 4° C and the protein concentrations were measured. Protein samples were thereafter combined with 4x Laemmli sample buffer (Boston BioProducts, Ashland, MA), denatured, and separated by SDS-PAGE. Following transfer to an Amersham Hybond-P membrane (GE Heathcare, Piscataway, NJ), the membrane was blocked and probed with Gal-9 (Novus Biologicals, Littleton CO), Tim-3 (R&D, Mineapdis, MN), phospho-STAT-1 (Tyr701), phospho-STAT-3 (Tyr705) or total STAT-1, STAT-3 antibody (Cell Signaling Technology, Inc, Danvers, MA) or β-actin (Santa Cruz) at 4° C overnight. Finally, the membrane was incubated with a horseradish peroxidase (HRP)-conjugated secondary antibody (Millipore, Temecula, CA) and developed by Amersham^TM^ ECL Plus Western Blotting Detection Reagents (GE Healthcare Biosciences, Pittsburgh, PA) on Kodak X-OMAT-LS X-ray film (Sigma-Aldrich, St. Louis, MO). Specific bands were quantified by densitometry.

To detect the physical association of Tim-3/Gal-9, 1 x 10^7^ purified M/M_Φ_ were stimulated with or without 1 µg/ml of LPS and 2.5 µg/ml of R848 for 6 h, and cell lysates were prepared following Pierce^®^ co-immunoprecipitation (ip) kit (Thermo Scientific, Rockford, IL) instructions. Samples were incubated with 3 µg of anti-Gal-9 or IgG antibodies (Novus Biologicals, Littleton CO) overnight at 4° C, followed by additional 2 h of incubation with 20 ml of Protein A/G PLUS-Agarose (Santa Cruz Biotechnology, Santa Cruze, CA) at 4° C. After washing with lysis buffer, the samples were denatured with Laemmli sample buffer (Boston BioProducts, Ashland, MA) and separated by SDS-PAGE. Following transfer to Amersham Hybond-P membrane (GE Heathcare, Piscataway, NJ), the membrane was blocked by 2.5% BSA-PBS and probed with anti-Tim-3 antibody (F38-2E2, BioLegend, San Diego, CA) at 4° C overnight. β-actin (Santa Cruz Biotechnology, Santa Cruz, CA) was used to probe the cell lysates for equal protein input. The HRP-secondary antibody probe and specific band detection were carried out as described above. To inhibit random Gal-9 bound to Tim-3 during the procedure^19^, α-lactose (30 mM, Sigma), a competitive substrate that inhibits the random interaction between galectins and their receptors, was added to the TLR-stimulated M/M_Φ_ cell lysates prior to co-ip experiments.

### Statistical analysis

Study results are summarized for each group and results are expressed as the mean ± standard error of mean from duplicates of each specific sample (SE). Comparison between two groups was performed by multiple comparisons testing/least significant difference on the ANOVA Prism software (version 4; GraphPad Software). Values of p<0.05 (*), p<0.01(**), p<0.001 (***) were considered significant or very significant. NS = no significance.

## Supporting Information

Figure S1 **Tim-3 and Gal-9 expressions by THP-1 or monocytes with or without Gal-9 transfection and/or TLR stimulation.** A) Intracellular Gal-9 expression in THP-1 cells following Gal-9 or control plasmid transfection, with or without TLR stimulation, was detected by flow cytometric analysis. Representative dot plots and summary data from repeated experiments were shown. B) Extracellular Gal-9 secretion into the supernatant of THP-1 cells following Gal-9 or control plasmid transfection, with or without TLR stimulation, was detected by ELISA. Summary data from repeated experiments were shown. C) Tim-3 cell surface expression on CD14^+^ monocytes, with or without LPS stimulation in the presence or absence of anti-human CD284 (TLR4 blocking antibody), was detected by flow cytometry. Representative dot plots with percentage of cell frequencies in the gated area were shown.
(TIF)

Figure S2 **Tim-3 and Gal-9 localization/co-localization in resting and TLR-stimulated M/M_Ø_ detected by immunofluorescent microscopy.** Purified M/M_Ø_ were stimulated with LPS/R848 for 0, 1, 2, 3, 6 h; after fixation/permeabilization, intracytoplasmic Tim-3 (green) and Gal-9 (red) staining as well as DAPI nuclear staining (blue), isotype control staining, and their imaging merges were observed by immunofluorescent microscopy as described in the Methods. A typical cell with Tim-3/Gal-9 imaging merge (yellow) as evidence of their co-localization in M/M_Ø_ is denoted in the square. Magnification 40x for all panels with scale bar = 50 µm.
(TIF)
