# Supplementary figures and images for: Cis Association of Galectin-9 with Tim-3 Differentially Regulates IL-12/IL-23 Expressions in Monocytes via TLR Signaling
Source: PLoS One. 2013 Aug 14;8(8):e72488. doi: 10.1371/journal.pone.0072488 (PMC3743775; doi:10.1371/journal.pone.0072488)

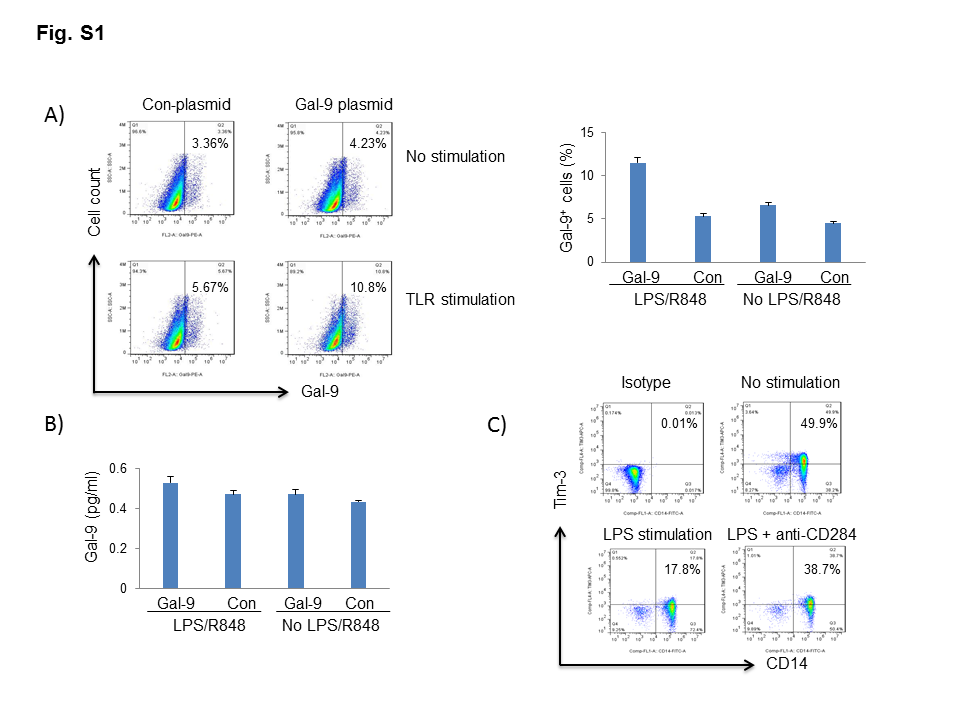

Supplement: Figure S1 — Tim-3 and Gal-9 expressions by THP-1 or monocytes with or without Gal-9 transfection and/or TLR stimulation. A) Intracellular Gal-9 expression in THP-1 cells following Gal-9 or control plasmid transfection, with or without TLR stimulation, was detected by flow cytometric analysis. Representative dot plots and summary data from repeated experiments were shown. B) Extracellular Gal-9 secretion into the supernatant of THP-1 cells following Gal-9 or control plasmid transfection, with or without TLR stimulation, was detected by ELISA. Summary data from repeated experiments were shown. C) Tim-3 cell surface expression on CD14+ monocytes, with or without LPS stimulation in the presence or absence of anti-human CD284 (TLR4 blocking antibody), was detected by flow cytometry. Representative dot plots with percentage of cell frequencies in the gated area were shown. (TIF) [file pone.0072488.s001.tif]

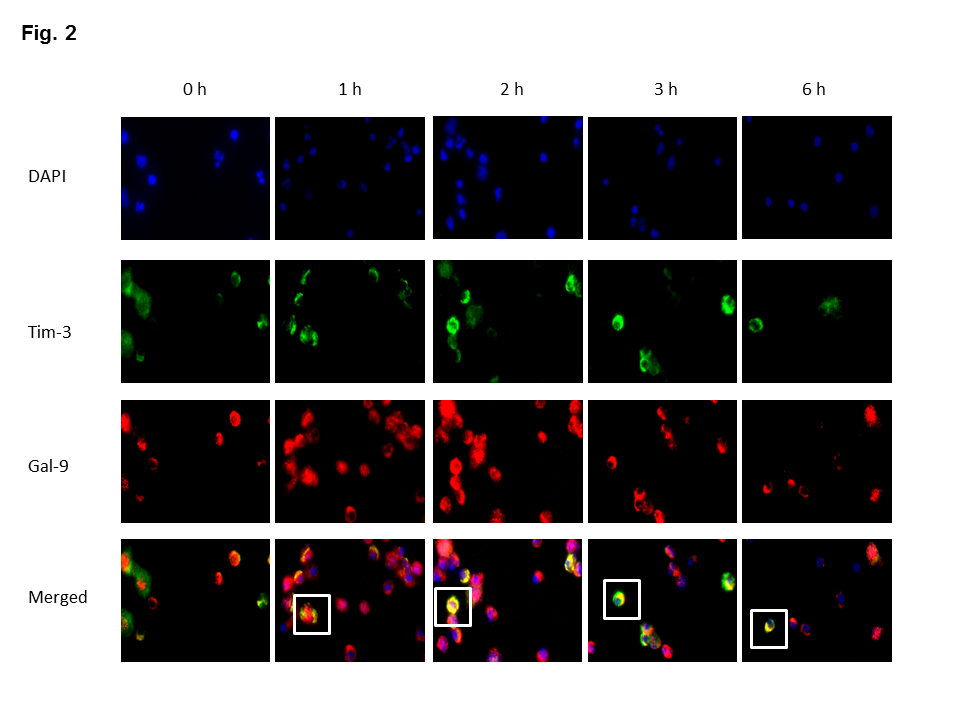

Supplement: Figure S2 — Tim-3 and Gal-9 localization/co-localization in resting and TLR-stimulated M/MØ detected by immunofluorescent microscopy. Purified M/MØ were stimulated with LPS/R848 for 0, 1, 2, 3, 6 h; after fixation/permeabilization, intracytoplasmic Tim-3 (green) and Gal-9 (red) staining as well as DAPI nuclear staining (blue), isotype control staining, and their imaging merges were observed by immunofluorescent microscopy as described in the Methods. A typical cell with Tim-3/Gal-9 imaging merge (yellow) as evidence of their co-localization in M/MØ is denoted in the square. Magnification 40x for all panels with scale bar = 50 µm. (TIF) [file pone.0072488.s002.tif]
